# Determination of FVIIa-sTF Inhibitors in Toxic *Microcystis* Cyanobacteria by LC-MS Technique

**DOI:** 10.3390/md14010007

**Published:** 2015-12-30

**Authors:** Andrea Roxanne J. Anas, Anna Nakajima, Chiaki Naruse, Mineka Tone, Hirohiko Asukabe, Ken-ichi Harada

**Affiliations:** 1 Faculty of Pharmacy, Meijo University, Tempaku, Nagoya 468-8503, Japan; 2 Graduate School of Environmental and Human Sciences, Meijo University, Tempaku, Nagoya 468-8503, Japan; g0973345@ccalumni.meijo-u.ac.jp (A.N); 100973149@ccalumni.meijo-u.ac.jp (C.N.); 110973437@ccalumni.meijo-u.ac.jp (M.T.); asukabe@bj8.so-net.ne.jp (H.A.); 3 Department of Brain Functions, Division of Stress Adaptation and Protection, Research Institute of Environmental Medicine, Nagoya University, Nagoya 464-8601, Japan

**Keywords:** cyanobacteria, toxic *Microcystis*, anticoagulant, fVIIa-sTF inhibitors, peptides, aeruginosins, blood coagulation cascade

## Abstract

The blood coagulation cascade involves the human coagulation factors thrombin and an activated factor VII (fVIIa). Thrombin and fVIIa are vitamin-K-dependent clotting factors associated with bleeding, bleeding complications and disorders. Thrombin and fVIIa cause excessive bleeding when treated with vitamin-K antagonists. In this research, we explored different strains of toxic *Microcystis aeruginosa* and cyanobacteria blooms for the probable fVIIa-soluble Tissue Factor (fVIIa-sTF) inhibitors. The algal cells were subjected to acidification, and reverse phase (ODS) chromatography-solid phase extraction eluted by water to 100% MeOH with 20%-MeOH increments except for *M. aeruginosa* NIES-89, from the National Institute for Environmental Studies (NIES), which was eluted with 5%-MeOH increments as an isolation procedure to separate aeruginosins 89A and B from co-eluting microcystins. The 40%–80% MeOH fractions of the cyanobacterial extract are active against fVIIa-sTF. The fVIIa-sTF active fractions from cultured cyanobacteria and cyanobacteria blooms were subjected to liquid chromatography-mass spectrometry (LC-MS). The 60% MeOH fraction of *M. aeruginosa* K139 exhibited an *m/z* 603 [M + H]^+^ attributed to aeruginosin K139, and the 40% MeOH fraction of *M. aeruginosa* NIES-89 displayed ions with *m/z* 617 [M − SO_3_ + H]^+^ and *m/z* [M + H]^+^ 717, which attributed to aeruginosin 89. Aeruginosins 102A/B and 298A/B were also observed from other toxic strains of *M. aeruginosa* with positive fVIIa-sTF inhibitory activity. The active fractions contained cyanobacterial peptides of the aeruginosin class as fVIIa-sTF inhibitors detected by LC-MS.

## 1. Introduction

The blood coagulation cascade [1,2,3,4,5] is composed of intrinsic, extrinsic and common pathways involving human coagulation factors. It is initiated by vascular injury and tissue factor (TF) exposure, which triggers the extrinsic pathway [2]. The extrinsic pathway involves activated factor VII-tissue factor (fVIIa-TF) complex activated by Ca^2+^, cephalin or phospholipid [6]. The activation of the fVIIa-TF complex triggers activation of factor X (fX) to activated factor X (fXa) leading to activation of activated factor II (fIIa) or thrombin generation [2]. Thrombin generation needs fVIIa-TF complex, which initiates coagulation and has become the target of therapeutic studies [7]. The activated factor VII (fVIIa) as a vitamin K-dependent clotting factor, when complexed with tissue factor (TF), activates fXa and fIIa (thrombin) [1,2,3,4]. The vitamin-K-dependent clotting factors IIa (thrombin) and fVIIa are linked to inherited bleeding disorders and complications causing clinical and acquired problems [8]. The vitamin-K-dependent clotting factors could be treated with coumarin or warfarin, which acts as vitamin K antagonists (VKAs) [9]. However, warfarin as a VKA employs excessive bleeding and bleeding complications through the years [9]. The VKAs offer some problems when taken orally, e.g., narrow therapeutic window [9].

The cyanobacteria from freshwater and terrestrial environments are the new treasure troves for drug discovery [10]. They are the promising sources of serine protease inhibitors, cytotoxic metabolites, and antimicrobials [10]. The serine protease inhibitors are mostly peptides of either cyclic or linear structure. These peptides with serine protease inhibitory activities are comprised of anabaenopeptins [11], aeruginosins [12,13,14], micropeptins [14,15,16,17], aeruginopeptins [18], and other peptides. The cyanobacterial peptides with serine protease inhibitory activities could be explored as scaffolds or anticoagulants in the blood coagulation cascade [1,2,3,4,19]. In our review [19], we have hypothesized that the toxic *Microcystis* cyanobacteria are potent sources of fVIIa-sTF inhibitors. The *Microcystis* contains toxic microcystins and its associated non-toxic peptides [18]. Mostly, these non-toxic peptides give significant serine protease inhibitory properties, which could be applied as anticoagulants for enzymes in the blood coagulation cascade [1,2,3,4], and could minimize bleeding and bleeding complications [6,20,21]. The serine protease inhibitors biosynthesis by *Microcystis* strains have promising thrombin, plasmin, and trypsin inhibitory activities, and could be used as anticoagulants of the blood coagulation cascade [19]. We have identified or hypothesized some scaffolds responsible for inhibition against fVIIa-sTF [19]. In this study, we have explored toxic *Microcystis* using the tandem liquid chromatography-mass spectrometry (LC-MS) technique to identify the potent fVIIa-sTF inhibitors. This research deals with the identification of potent fVIIa-sTF inhibitors from toxic *Microcystis* cyanobacteria using the technique above.

## 2. Results and Discussion

**Figure 1:**
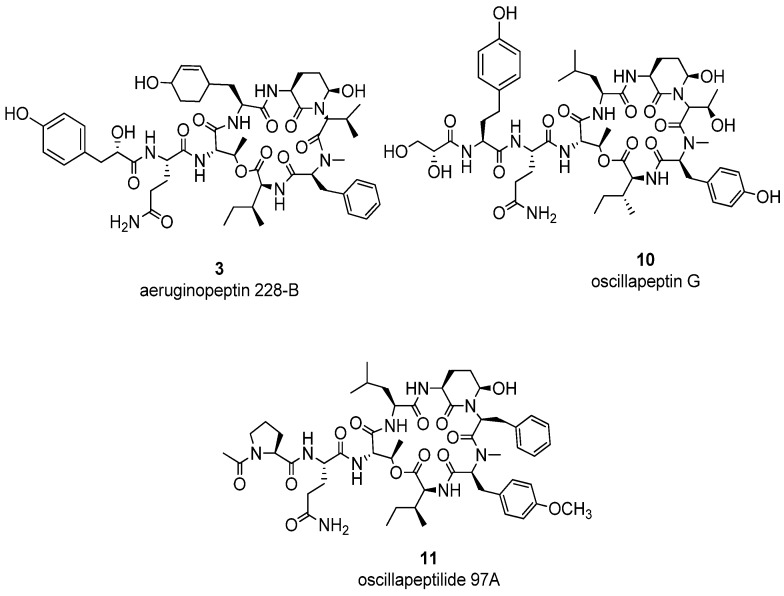
Compounds active against fVIIa.

Peptide compounds **1**–**25** (Table 1) previously isolated in our laboratory, like aeruginopeptins, anabaenopeptins, anabaenopeptilides, and microcystins, were tested in thrombin, fVIIa, and fVIIa-sTF inhibitory assays. All of the tested compounds did not inhibit thrombin except spumigins A (**21**) and J (**22**) [22]. Compounds **21** and **22** were active at 100 µg/mL and 10 µg/mL, respectively, after a long-term storage. The three compounds (Figure 1), aeruginopeptin 228-B (**3**), oscillapeptin G (**10**) and oscillapeptilide 97A (**11**) were active against fVIIa with slow binding and inhibition from three to six hours at 10 and 1 µg/mL with l-α-cephalin buffer, and without soluble tissue factor (sTF). The sTF improved the activation of fVII to fVIIa in the experiment [23]. The sTF was used as a cofactor and an activator of fVIIa, with the presence of Ca^2+^ and cephalin or 3-*sn*-phosphatidylethanolamine. In the experiment, the sTF was utilized rather than TF because our target was fVIIa inhibition and not TF inhibition. Moreover, all compounds displayed no inhibition when further tested for fVIIa-sTF inhibitory assay. As a consequence, we explored 50 cyanobacteria strains, specifically toxic *Microcystis* and *Anabaena*, for the probable fVIIa-sTF inhibitory activities. Several cyanobacterial fractions processed in our laboratory (Table 2) gave promising fVIIa-sTF inhibitory activities. The *Microcystis aeruginosa* NIES-89, K139, M228, TAC 95 (H-strain), NIES-102, NIES-103, NIES-107, NIES-1025, NIES-1058, NIES-1071, NIES-1085, NIES-1099, NIES-1133, NIES-1043, and NIES-298 were found to be active in the fVIIa-sTF inhibitory assays. The 40%–80% MeOH fractions, with 40% and 60% MeOH as the active ones, presented a potent fVIIa-sTF activity at 100 µg/mL and 10 µg/mL for thrombin and fVIIa-sTF. The fVIIa-sTF assay was pursued in the screening of cyanobacterial extracts instead of fVIIa assay since in the human system, fVII partially existed as a complex of fVIIa-TF than fVIIa alone [23].

**Table 1:** Peptide compounds tested for fVII, fVIIa-sTF and thrombin assays.

Structural Class	Compound Name	Serine Protease Inhibitory Assays (µg/mL)	
1. Aeruginopeptins	95-A (**1**) 95-B (**2**)	*thrombin*, 10, 1:	(−)
		*fVIIa*, 100, 10:	(−)
		*fVIIa-sTF*, 100, 10, 1:	(−)
	228-B (**3**)	*thrombin*, 10, 1:	(−)
		*fVIIa*, 100, 10:	(+)
		*fVIIa-sTF*, 100, 10, 1:	(−)
	917S-A (**4**)	*thrombin*, 10:	(−)
	917S-B (**5**)	*fVIIa*, 100, 10:	(−)
	917S-C (**6**)	*fVIIa-sTF*, 10, 1:	(−)
2. Anabaenopeptins	A (**7**)	*thrombin*, 10:	(−)
	B (**8**)	*fVIIa*, 100, 10:	(−)
	C (**9**)	*fVIIa-sTF*, 10, 1:	(−)
	oscillapeptin G (**10**) oscillapeptilide 97A (**11**)	*thrombin*, 10, 1:	(−)
		*fVIIa*, 100, 10:	(+)
		*fVIIa-sTF*, 100, 10, 1:	(−)
	oscillapeptilide 97B (**12**)	*thrombin*, 10, 1:	(−)
		*fVIIa*, 100, 10:	(−)
		*fVIIa-sTF*, 100, 10, 1:	(−)
3. Anabaenapeptilides	90-A (**13**) 90-B (**14**)	*thrombin*, 10, 1:	(−)
		*fVIIa*, 100, 10:	(−)
		*fVIIa-sTF*, 100, 10, 1:	(−)
	202-A (**15**) 202-B (**16**)	*thrombin*, 10:1:	(−)
		*fVIIa*, 100, 10:	(−)
		*fVIIa-sTF*, 100, 10, 1:	(−)
	oscillamide Y (**17**)	*thrombin*, 10, 1:	(−)
		*fVIIa*, 100, 10:	(−)
		*fVIIa-sTF*, 10, 1:	(−)
4. Microcystins	LR (**18**)	*fVIIa-sTF*, 100, 10, 1:	(−)
	RR (**19**)	*fVIIa-sTF*, 100, 10, 1:	(−)
	YR (**20**)	*fVIIa-sTF*, 100, 10, 1:	(−)
5. Spumigins	A (**21**)	*thrombin*: 100, 10, 1:	(+,−,−)
		*fVIIa*, 100, 10:	(−)
		*fVIIa-sTF*, 100, 10, 1:	(−)
	J (**22**)	*thrombin*: 10, 1:	(+)
		*fVIIa*, 100, 10:	(−)
		*fVIIa-sTF*, 100, 10, 1:	(−)
6. Other Peptides	nostophycin (**23**)	*thrombin,* 10:	(−)
		*fVIIa*, 100, 10:	(−)
		*fVIIa-sTF*, 10, 1:	(−)
	microcyclamide (**24**) microviridin (**25**)	*thrombin*, 10, 1:	(−)
		*fVIIa*, 100, 10:	(−)
		*fVIIa-sTF*, 10, 1:	(−)

**Table 2:** LC-MS data of *M. aeruginosa* thrombin and fVIIa-sTF active fractions.

No.	*M. aeruginosa* Strain	% MeOH Fraction	Retention Time,t*_R_* (min)	(*m/z)*	Ions Detected	Compounds Detected
1	NIES-89	40	8.3	637.89	[M − SO_3_ + H]^+^	aeruginosin 89A/B (**26**/**27**)
			8.3	717.60	[M + H]^+^	(**26**/**27**)
			8.8	638.05	[M − SO_3_ + H]^+^	(**26**/**27**)
			8.8	717.63	[M + H]^+^	(**26**/**27**)
			9.8	638.07	[M − SO_3_ + H]^+^	(**26**/**27**)
			10.2	638.03	[M − SO_3_ + H]^+^	(**26**/**27**)
		45	8.3	637.81	[M − SO_3_ + H]^+^	aeruginosin 89A/B (**26**/**27**)
			8.3	717.68	[M + H]^+^	(**26**/**27**)
			9.9	637.91	[M − SO_3_ + H]^+^	(**26**/**27**)
			9.9	717.40	[M + H]^+^	(**26**/**27**)
			18.9	996.05	[M + H]^+^	microcystin-LR (**18**)
2	K139	60	5.1	603.50	[M + H]^+^	aeruginosin K139 (**30**)
			5.1	621.39	[M + H_2_O + H]^+^	(**30**)
			6.2	603.34	[M + H]^+^	(**30**)
			7.6	603.33	[M + H]^+^	(**30**)
			8.4	603.34	[M + H]^+^	(**30**)
3	M228	60	5.2	605.48	[M + H]^+^	aeruginosin 298A (**32**)
			6.3	621.34	[M + H_2_O + H]^+^	aeruginosin K139 (**30**)
			7.7	603.41	[M + H]^+^	(**30**)
			8.5	603.35	[M + H]^+^	(**30**)
			14.9	1031.91	[M + H − H_2_O]^+^	aeruginopeptin 228-B (**3**)
			14.9	1049.43	[M + H]^+^	(**3**)
			15.5	1028.27	[M + H − H_2_O]^+^	aeruginopeptin 228-A (**33**)
			18.4	1046.05	[M + H]^+^	microcystin-YR (**20**)
4	TAC 95	40	5.5	605.65	[M + H]^+^	aeruginosin 298A (**32**)
			5.5	621.61	[M + H_2_O + H]^+^	aeruginosin K139 (**30**)
			6.5	603.61	[M + H]^+^	(**30**)
			7.8	603.78	[M + H]^+^	(**30**)
			8.5	603.77	[M + H]^+^	(**30**)
		60	7.7	603.62	[M + H]^+^	aeruginosin K139 (**30**)
			8.5	603.59	[M + H]^+^	(**30**)
			8.8	603.92	[M + H]^+^	(**30**)
			15.1	1132.92	[M + H − H_2_O]^+^	aeruginopeptin 95B (**2**)
			15.4	1129.04	[M + H − H_2_O]^+^	aeruginopeptin 95A (**1**)
5	NIES-102	40	1.8	653.86	[M − SO_3_ + H]^+^	aeruginosin 102A/B (**34**/**35**)
			1.8	733.71	[M + H]^+^	(**34**/**35**)
			3.0	653.94	[M − SO_3_ + H]^+^	(**34**/**35**)
			3.0	733.69	[M + H]^+^	(**34**/**35**)
			3.5	653.96	[M − SO_3_ + H]^+^	(**34**/**35**)
			3.5	733.60	[M + H]^+^	(**34**/**35**)
6	NIES-103	40	4.9	653.36	[M − SO_3_ + H]^+^	aeruginosin 102A/B (**34**/**35**)
			4.9	733.15	[M + H]^+^	(**34**/**35**)
		60	5.3	653.37	[M − SO_3_ + H]^+^	aeruginosin 102A/B (**34**/**35**)
			5.3	733.07	[M + H]^+^	(**34**/**35**)
			16.0	520.14	[M + 2H]^2+^	microcystin-RR (**19**)
			16.0	1038.61	[M + H]^+^	(**19**)
			18.6	523.39	[M + 2H]^2+^	microcystin-YR(**20**)
			18.6	1045.62	[M + H]^+^	(**20**)
			19.0	995.65	[M + H]^+^	microcystin-LR (**18**)
7	NIES-107	60	7.9	606.28	[M + H]^+^	aeruginosin 298A (**32**)
			15.9	520.10	[M + 2H]^2+^	microcystin-RR (**19**)
			15.9	1038.58	[M + H]^+^	(**19**)
			19.0	995.64	[M + H]^+^	microcystin-LR (**18**)
8	NIES-1025	40	2.3	653.33	[M − SO_3_ + H]^+^	aeruginosin 102A/B (**34**/**35**)
			4.9	733.14	[M + H]^+^	(**34**/**35**)
		60	5.0	653.34	[M − SO_3_ + H]^+^	aeruginosin 102A/B (**34**/**35**)
			5.0	733.13	[M + H]^+^	(**34**/**35**)
			15.8	520.15	[M + 2H]^2+^	microcystin-RR (**19**)
			15.8	1038.71	[M + H]^+^	microcystin-RR (**19**)
			19.0	995.71	[M + H]^+^	microcystin-LR (**18**)
		80	5.3	653.32	[M − SO_3_ + H]^+^	aeruginosin 102 (**34**/**35**)
			5.3	733.18	[M + H]^+^	(**34**/**35**)
			15.7	520.14	[M + 2H]^2+^	microcystin-RR (**19**)
			15.7	1038.71	[M + H]^+^	(**19**)
			19.0	995.69	[M + H]^+^	microcystin-LR (**18**)
			22.5	1029.71	[M + H]^+^	microcystin-FR (**29**)
9	NIES-1058	40	9.0	717.07	[M + H]^+^	aeruginosin 89A/B (**26**/**27**)
			10.7	717.07	[M + H]^+^	(**26**/**27**)
		60	8.9	717.13	[M + H]^+^	aeruginosin 89A/B (**26**/**27**)
			10.9	717.13	[M + H]^+^	(**26**/**27**)
			15.9	520.15	[M + 2H]^2+^	microcystin-RR (**19**)
			15.9	1038.60	[M + H]^+^	(**19**)
			18.5	1045.60	[M + H]^+^	microcystin-YR (**20**)
10	NIES-1071	60	8.5	637.40	[M − SO_3_ + H]^+^	aeruginosin 89A/B (**26**/**27**)
			8.5	717.12	[M + H]^+^	(**26**/**27**)
			9.0	637.35	[M − SO_3_ + H]^+^	(**26**/**27**)
			9.0	717.11	[M + H]^+^	(**26**/**27**)
			10.1	637.33	[M − SO_3_ + H]^+^	(**26**/**27**)
			10.1	717.09	[M + H]^+^	(**26**/**27**)
			10.4	637.33	[M − SO_3_ + H]^+^	(**26**/**27**)
			10.4	717.07	[M + H]^+^	(**26**/**27**)
			15.8	513.09	[M + 2H]^2+^	7-desmethylmicrocystin-RR(**28**)
			15.8	1024.64	[M + H]^+^	(**28**)
			15.9	520.12	[M + 2H]^2+^	microcystin-RR (**19**)
			15.9	1038.60	[M + H]^+^	microcystin-RR (**19**)
			19.0	995.61	[M + H]^+^	microcystin-LR (**18**)
11	NIES-1085	60	9.5	637.33	[M − SO_3_ + H]^+^	aeruginosin 89A/B (**26**/**27**)
			11.1	717.18	[M + H]^+^	(**26**/**27**)
12	NIES-1099	60	8.4	637.44	[M − SO_3_ + H]^+^	aeruginosin 89A/B (**26**/**27**)
			8.4	717.09	[M + H]^+^	(**26**/**27**)
			10.0	637.38	[M − SO_3_ + H]^+^	(**26**/**27**)
			10.0	717.11	[M + H]^+^	(**26**/**27**)
			10.3	637.36	[M − SO_3_ + H]^+^	(**26**/**27**)
			10.3	717.14	[M + H]^+^	(**26**/**27**)
			15.8	520.11	[M + 2H]^2+^	microcystin-RR (**19**)
			15.8	1038.60	[M + H]^+^	(**19**)
			18.4	1045.58	[M + H]^+^	microcystin-YR(**20**)
13	NIES-1133	40	1.8	653.41	[M − SO_3_ + H]^+^	aeruginosin 102A/B (**34**/**35**)
			1.8	733.14	[M + H]^+^	(**34**/**35**)
			2.3	653.44	[M − SO_3_ + H]^+^	(**34**/**35**)
			2.3	733.13	[M + H]^+^	(**34**/**35**)
			3.2	653.37	[M − SO_3_ + H]^+^	(**34**/**35**)
			3.2	733.14	[M + H]^+^	(**34**/**35**)
			3.7	653.39	[M − SO_3_ + H]^+^	(**34**/**35**)
			3.7	733.11	[M + H]^+^	(**34**/**35**)
			4.0	653.35	[M − SO_3_ + H]^+^	(**34**/**35**)
			4.0	733.09	[M + H]^+^	(**34**/**35**)
		60	9.0	637.39	[M − SO_3_ + H]^+^	aeruginosin 89A/B (**26**/**27**)
			9.9	637.39	[M − SO_3_ + H]^+^	(**26**/**27**)
			9.9	717.34	[M + H]^+^	(**26**/**27**)
			15.6	520.16	[M + 2H]^2+^	microcystin-RR (**19**)
			15.6	1038.56	[M + H]^+^	(**19**)
			18.9	498.44	[M + 2H]^2+^	microcystin-LR (**18**)
			18.9	995.70	[M + H]^+^	(**18**)
14	NIES-1043	40	6.4	733.12	[M + H]^+^	aeruginosin 102A/B (**34**/**35**)
			10.4	717.03	[M + H]^+^	aeruginosin 89A/B (**26**/**27**)
			10.4	733.86	[M + H]^+^	(**34**/**35**)
15	NIES-298	40	7.1	605.47	[M + H]^+^	aeruginosin 298A (**32**)
			7.1	621.40	[M + H_2_O + H]^+^	aeruginosin K139 (**30**)
		60	6.6	603.37	[M + H]^+^	aeruginosin K139 (**30**)
			7.4	621.40	[M + H_2_O + H]^+^	(**30**)
		80	6.6	603.35	[M + H]^+^	aeruginosin K139 (**30**)
			6.6	621.38	[M + H_2_O + H]^+^	(**30**)

The 40%–65% MeOH fractions of *M. aeruginosa* NIES-89 extract contained mostly aeruginosins 89A (**26**) and B (**27**) with accompanying microcystins-LR (**18**) with mass to charge (*m/z*) 996 [M + H]^+^ at a retention time (t*_R_*) 18.9 min (Table 2). Microcystins were also found in *M. aeruginosa* M228 in the form of microcystin-YR (**20**) t*_R_* 18.4 min. In addition, *M. aeruginosa* NIES-103 contained microcystins-LR (**18**) with *m/z* 995 [M + H]^+^ at t*_R_* 19.0 min, -RR (**19**) with *m/z* 520 [M + 2H]^2+^ and *m/z* 1038 [M + H]^+^ at t*_R_* 16.0 min, and -YR (**20**) with *m/z* 523 [M + 2H]^2+^ and *m/z* 1045 [M + H]^+^ at t*_R_* 18.6 min. Compounds **18** and **19** could also be observed in 60% MeOH fraction of *M. aeruginosa* NIES-1133. Furthermore, compounds **18** and **19** could also be found in *M. aeruginosa* NIES-107–60% MeOH fraction, and NIES-1025–60% to 80% MeOH fractions with accompanying microcystin-FR (**29**) at t*_R_* 22.5 min with *m/z* 1029 [M + H]^+^. Compounds **19** and **20** could also be found in 60% MeOH fractions of *M. aeruginosa* NIES-1058 and NIES-1099. The *M. aeruginosa* NIES-1071 contained microcystins-LR (**18**), -RR (**19**) and 7-desmethylmicrocystin RR (**28**) at t*_R_* 15.8 min with *m/z* 513 [M + 2H]^2+^ and 1024 [M + H]^+^. Thus, we tested several microcystins (**18**–**20**) for inhibition of fVIIa-sTF complex (Table 1). However, microcystins-LR (**18**), -RR (**19**), and -YR (**20**) were not active against fVIIa-sTF. Further analysis of the active fractions by LC-MS, specifically *M. aeruginosa* K139 [24] and NIES-89 [13], led to the identification of the aeruginosins as the active compounds.

**Figure 2:**
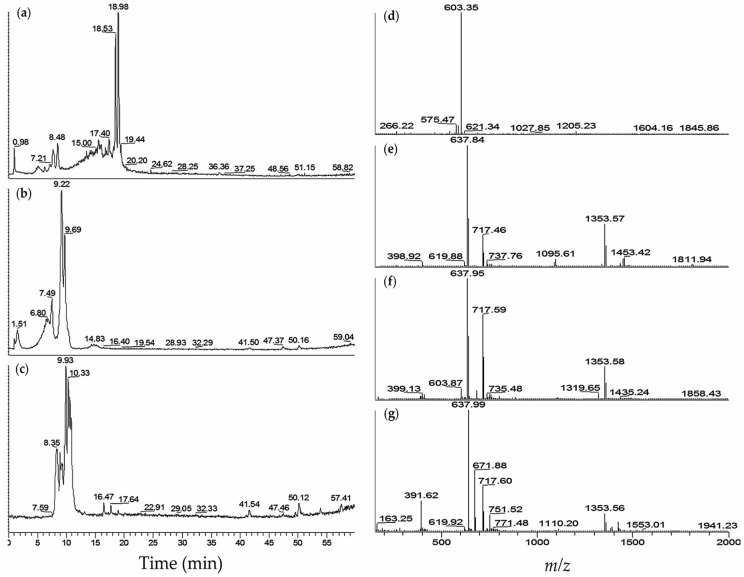
LC-MS profiles of *M. aeruginosa* K139 and NIES-89 cyanobacteria fVIIa-sTF active fractions: (**a**) Total ion chromatogram (TIC) of *M. aeruginosa* K139–60% MeOH; (**b**) TIC of *M. aeruginosa* NIES-89–40% MeOH; (**c**) TIC of *M. aeruginosa* NIES-89–45% MeOH; (**d**) Mass spectrum (MS) of *M. aeruginosa* K139–60% MeOH, retention time (t*_R_*) 7.3–8.5 min; (**e**) MS of *M. aeruginosa* NIES-89–40% MeOH, t*_R_* 8.8–9.7 min; (**f**) MS of *M. aeruginosa* NIES-89–40% MeOH, t*_R_* 6.0–7.9 min; (**g**) MS of *M. aeruginosa* NIES-89–45% MeOH, t*_R_* 7.9–11.3 min.

Analysis of the fVIIa-sTF active extracts of *M. aeruginosa* K139 and NIES-89 by LC-MS [25] gave a good lead for the active compounds present as fVIIa-sTF inhibitors (Figure 2, Table 2). LC-MS analysis of *M. aeruginosa* K139–60% MeOH fraction identified aeruginosin K139 (**30**) [24] as the active component with *m/z* 603 [M + H]^+^ at t*_R_* 5.1–8.4 min (Figure 2a,d). An *m/z* 621 [M + H_2_O + H]^+^ at t*_R_* 5.1 min was also found in the MS spectrum as a diagnostic for aeruginosin K139 (**30**) (Table 2). Aeruginosin K139 (**30**) was also noted in other toxic *M. aeruginosa* strains with similarly observed *m/z* as *M. aeruginosa* K139. The *M. aeruginosa* M228, NIES-298, and TAC 95 (H-strain)–40% and 60% MeOH fractions gave similar characteristic patterns as *M. aeruginosa* K139. However, ions with *m/z* 605 [M + H]^+^ at t*_R_* 5.2 min, and *m/z* 1028 [M + H − H_2_O]^+^ at t*_R_* 15.5 min were assigned as aeruginosin 298A (**32**) and aeruginopeptin 228-A (**33**), respectively.

We have isolated aeruginosin K139 (**30**) but unfortunately, complete chemical shift assignments were not determined [26]. The paper by Nishizawa *et al.* [24] published aeruginosin K139 (**30**) chemical structure by MS elucidation. However, the stereochemistry of the compound was not deduced. Aeruginosin K139 (**30**) will be elucidated completely in our next paper. Moreover, aeruginosin K139 (**30**) has a chemical structure similar to aeruginosin 602 (**31**) reported by Welker *et al.* [27]. Aeruginosins K139 (**30**) and 602 (**31**) have identical fragmentation pattern reported by Nishizawa *et al.* [24] and Welker *et al.* [27]. Both compounds were also elucidated using the LC-MS technique. However, for consistency, this paper will refer aeruginosin with *m/z* 603 [M + H]^+^ as aeruginosin K139 (**30**), in which our group detected from *M. aeruginosa* K139. 

The 40%–45% MeOH extracts of *M. aeruginosa* NIES-89 contained a mixture of aeruginosins 89A/B (**26/27**) [13] with *m/z* 637 [M − SO_3_ + H]^+^ and *m/z* 717 [M + H]^+^ at t*_R_* 6.0–11.0 min (Figure 2b,c). Further analysis of *M. aeruginosa* NIES-89–40% MeOH by solvent optimization from 10% to 15% MeCN with 0.1% HCOOH over 60 min using reverse phase super ODS (100 × 2 mm) at 200 °C capillary temperature, exhibited tautomerization [13] of aeruginosins 89A (**26**) and B (**27**) with *m/z* 637 [M − SO_3_ + H]^+^and *m/z* 717 [M + H]^+^ at t*_R_* 30.4, 35.5, 44.2, and 47.3 min (data not shown).

We tested other toxic *Microcystis* strains for the presence of aeruginosins. Aeruginosins could also be found in some other strains of toxic *Microcystis*, with the presence of aeruginopeptins and microcystins. Indeed, the *M. aeruginosa* M228 strain was positive against fVIIa-sTF assay. The aeruginopeptins or microcystin-YR (**20**), with t*_R_* 14.9–18.4 min, co-existed with the active compounds. However, testing of the pure compounds of aeruginopeptins and microcystins (Table 1) for fVIIa-sTF assay proved that the potent compounds responsible for such activity were aeruginosins with the ions at *m/z* 603 [M + H]^+^ to *m/z* 621 [M + H_2_O + H]^+^. The dominant ions at *m/z* 603 [M + H]^+^ among positive fVIIa-sTF cyanobacterial extracts were attributed to aeruginosin K139 (**30**). The *m/z* 605 [M + H]^+^ was dereplicated as aeruginosin 298A (**32**). In addition, the LC-MS analyses of the fVIIa-sTF potent 40%–60% MeOH fractions of *M. aeruginosa* TAC 95, NIES-102 (Table 2) and other *M.* *aeruginosa* strains contained compounds belonging to the aeruginosin family. The *M. aeruginosa* TAC 95, 60% MeOH fraction, contained aeruginosin K139 (**30**) as an active compound with *m/z* 603 [M + H]^+^ at t*_R_* 7.7–8.8 min (Table 2). The aeruginopeptins 95A (**1**) and B (**2**) co-eluted with compound **30** in 60% MeOH fraction of TAC 95 strain. Compounds **1** and **2** eluted subsequently at t*_R_* 15.4 and 15.1, with *m/z* 1129 [M + H − H_2_O]^+^ and *m/z* 1132 [M + H − H_2_O]^+^, respectively. In addition, the aeruginosin 298A (**32**) eluted in 40% MeOH of the aforementioned cyanobacteria strain with t*_R_* 5.5 min. In *M. aeruginosa* NIES-102, aeruginosins 102A (**34**) and B (**35**) were present with the *m/z* 653 [M − SO_3_ + H]^+^ at t*_R_* 1.8–3.5 min. The presence of compounds **34** and **35** had extended through other strains of toxic *M. aeruginosa*, *i.e.*, NIES-103, NIES-1025, NIES-1133, and NIES-1043. Compounds **34** and **35** eluted at t*_R_* 1.8–6.4 min with *m/z* 653 [M − SO_3_ + H]^+^ and *m/z* 733 [M + H]^+^.

The LC-MS spectrum (Figure 2) of aeruginosins 89A (**26**) and B (**27**) matched with the data of Ishida *et al.* [13]. An *m/z* 637 [M − SO_3_ + H] was assigned as a desulfated ion, and with an observed [M + H]^+^ at *m/z* 717. An observed tautomerization of aeruginosins reported by Ishida’s group [13] was verified in the experiment. In Figure 2b, the 40% ODS MeOH fraction of *M. aeruginosa* NIES-89 displayed peaks at 6.8, 7.4, 9.2, and 9.6 min with ions *m/z* 637 [M − SO_3_ + H]^+^ and *m/z* 717 [M + H]^+^ (Figure 2e,f). An *m/z* 1353 [2M − SO_3_ + H]^+^ could also be observed in the spectrum.

**Figure 3:**
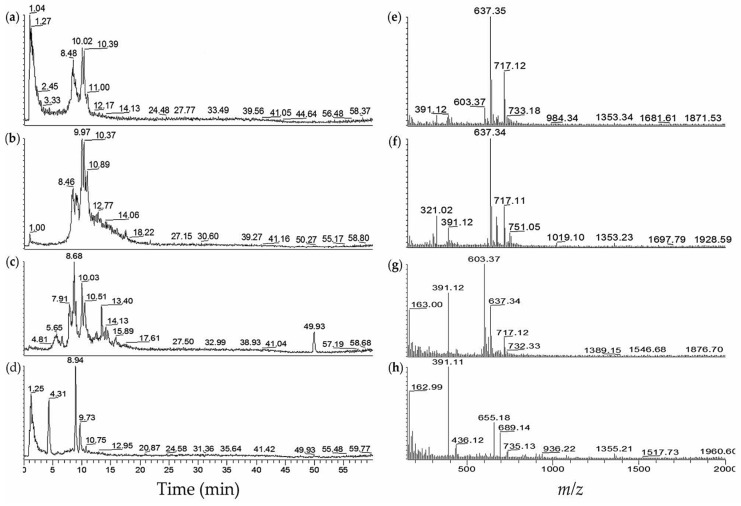
LC-MS profiles of fVIIa-sTF active cyanobacterial extracts from algal blooms. (**a**) Extracted ion chromatogram (EIC *m/z* 600–800) of JX-1-5 (from Ibaraki)–40% MeOH; (**b**) EIC *m/z* 600–800 of JX-1-5–60% MeOH; (**c**) EIC *m/z* 600–800 of Koyaike site 2–60% MeOH; (**d**) EIC *m/z* 600-800 of Koyaike site 3–40% MeOH; (**e**) ESI-full MS of JX-1-5–40% MeOH, t*_R_* 8.2–10.2 min; (**f**) ESI-full MS of JX-1-5–60% MeOH, t*_R_* 8.1–11.7 min; (**g**) ESI-full MS of Koyaike site 2–60% MeOH, t*_R_* 5.4–14.5 min; (**h**) ESI-full MS of Koyaike site 3–40% MeOH, t*_R_* 0.01–13.7 min.

Cyanobacteria blooms collected from Ibaraki and Hyogo, Japan were also processed in our laboratory. The 40%–60% MeOH fractions of JX-1-5 from Ibaraki, Japan were found to be positive in fVIIa-sTF assays. Analysis by LC-MS (Figure 3) showed the presence of aeruginosins 89A (**26**) and 89B (**27**) with the ions at *m/z* 637 [M − SO_3_ + H]^+^ and *m/z* 717 [M + H]^+^ at t*_R_* 8.2–10.2 min. The 60% MeOH fraction of Koyaike 2 from Hyogo contained both aeruginosins 89A/B (**26/27**) and K139 (**30**). The LC-MS chromatogram of 40% MeOH fraction (Koyaike 3) had retention times and expected *m/z* values similar to those of aeruginosins with the ions at *m/z* 655 to *m/z* 689.

The EC_50_s, calculated by Biodatafit [28], of the 40% MeOH fraction of *M. aeruginosa* NIES-89 containing aeruginosin 89A/B (**26**/**27**) were 0.010 µ*g*/mL and 7.123 µ*g*/mL for thrombin and fVIIa, respectively. Thus, the 40% MeOH fraction of *M. aeruginosa* NIES-89 had computed 0.001 thrombin/fVIIa ratio. The dual inhibitory activity of aeruginosins 89A/B (**26**/**27**), and also K139 (**30**), against thrombin and fVIIa enzymes, make aeruginosins good candidates for fVIIa-sTF inhibitors.

**Figure 4:**
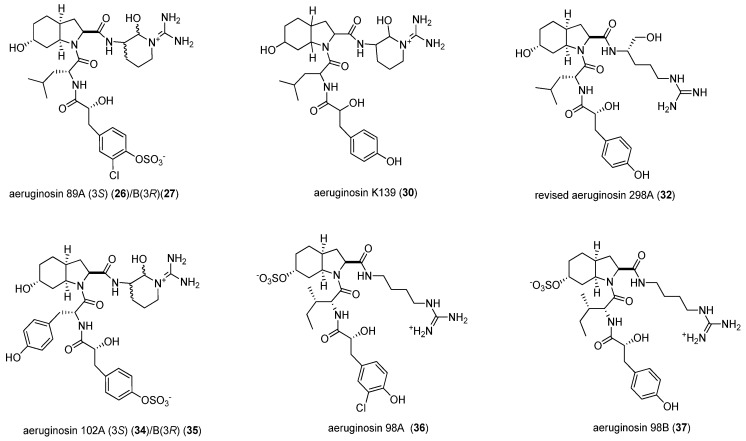
Aeruginosins detected by LC-MS.

We have detected aeruginosins 98A (**36**) and B (**37**) from *M. aeruginosa* NIES-98. The MeOH fractions from the aforementioned cyanobacteria are not active in the fVIIa-sTF assay. Thus, from our readings, we compare the fVIIa-sTF inhibitory activity of aeruginosins to phenylamidine. Kadono [29] has denoted the importance of phenylamidine P1 moiety in fVIIa inhibition, which has an inhibitory activity against fVIIa-sTF. The presence of the cyclic amino alcohol moiety in aeruginosins may contribute to efficient binding against fVIIa. However, this hypothesis needs to be established by a structure–activity relationship and subject to another paper. Based on Kadono’s paper [29], inhibitors “**1**–**5**” with linear structure and containing three peptide bonds exhibit both thrombin and fVIIa inhibitory activities. The number of peptide bonds contributes to the fVIIa inhibitory activity of the compounds and lessens its thrombin inhibition. The addition of one more peptide bond gives promising fVIIa-TF inhibitory activities. This additional peptide bond has been noted in inhibitors “**2**” to “**5**” [29] and aeruginosins. The presence of P3 moiety in aeruginosins has certain effects on inhibition of fVIIa and thrombin. The fVIIa and thrombin have the same catalytic triad Ser195-His58-Asp102, S1 pocket, and activation site Arg-Ile [30,31].

Similarly, the structure of aeruginosins could be compared to oscillarin from the cyanobacterium *Oscillatoria agardhii* [32,33] and dysinosins [33,34,35,36] from a sponge of the family *Dysideidae*. Both oscillarin and dysinosins have been reported as fVIIa and thrombin inhibitors [33].

Spumigins, similar to aeruginosins, also has thrombin inhibitory activities [22,37]. Both spumigins and aeruginosins are arginine-containing linear peptides. However, only the aeruginosins are active against fVIIa-sTF assay while spumigins A (**21**) and J (**22**) do not inhibit fVIIa and fVIIa-sTF. Both spumigins and aeruginosins contain a P1 side chain, which clings to thrombin’s specificity pocket containing Asp 189-engaged in ionic interactions with both classes of inhibitors [22,38].

Aeruginosins from toxic *Microcystis* cyanobacteria is a class of fVIIa-sTF inhibitors with thrombin-inhibiting activity. The aeruginosins could be developed into a specific fVIIa-sTF inhibitor that may avoid bleeding and bleeding complications. Some common fVIIa scaffolds from our review [19] have been identified, and we have correlated to the scaffolds of the cyanobacteria origin. The arginine and its arginine-derivatives (argininal and argininol) are essential for its fVIIa-sTF inhibition. In addition, structure–activity relationship (SAR) studies will be done in order to deduce the most active scaffold in aeruginosin. We hope to establish a particular SAR study between basic P1 arginine of aeruginosins and fVIIa enzyme. We will also consider the fVIIa enzyme and P3 moiety interaction as proposed in the study. Furthermore, synthesis and modifications have been deemed to make it specific for fVIIa. Assays involving a combination of co-factor(s) and enzymes (TF-fVIIa-fXa-fIIa, *etc.*) will be performed for a better diagnostic test for the specificity of aeruginosins.

## 3. Experimental Section

### 3.1. Culture Condition

Five- liter to ten-liter cyanobacterial cultures of 50 strains *M. aeruginosa* and *Anabaena* strains were grown in *M. aeruginosa* (MA) and C medium with N-Tris(hydroxymethyl) methyl-3-aminopropanesulfonic acid (TAPS) rather than Tris (hydroxymethyl) aminomethane (CT) media [39] for fVIIa-sTF and thrombin inhibitory assays. The *M. aeruginosa* K139 strain was grown in C medium with Bicine in preference for Tris (hydroxymethyl) aminomethane (CB medium) [24]. The *M. aeruginosa* strains were obtained from Microbial Culture Collection, National Institute for Environmental Studies (NIES), Japan unless otherwise indicated. The cultures were grown in a 5-L glass bottle by aeration at 20 °C for 2–4 weeks with continuous light except *M. aeruginosa* NIES-89 under 12L:12D cycle. The algal cells were centrifuged using Kubota 7000 centrifuge at 9000 rpm before lyophilization. The lyophilized cells were stored at −30 °C until micro-extraction.

### 3.2. Extraction

The freeze lyophilized algal cells (100 mg) were extracted with 3 mL (× 3) 5% acetic acid, homogenized for 30 min, and centrifuged using Kubota 5920 at 4000 rpm. The resulting supernate was evaporated *in vacuo* at 40 °C. The supernate was eluted by solid phase extraction (SPE) using Sep-Pak^®^ Vac 6 mL (1 g) C18/tC18 cartridge (*Waters brand*). Increasing concentrations of MeOH from water to 100% MeOH with 20% increments was used to elute the supernate. For *M. aeruginosa* NIES-89, a 5%-increment MeOH was used to separate aeruginosins from microcystins. The cyanobacterial extracts and pure peptides from *Microcystis* were subjected for *in vitro* assays. Standard microcystins were bought from Wako Pure Chemical Industries, Ltd., Osaka, Japan. The thrombin assay was performed following the procedure by Anas *et al.* [22,40,41], in parallel with fVIIa, fVIIa- sTF assays. The crude MeOH fractions active against fVIIa-sTF were subjected to LC-MS experiment to determine the active compounds present.

### 3.3. Serine Protease Inhibitory Assays

All assay experiments were done in a cold condition at 4 °C using an ice bucket until pre-incubation and reaction at 37 °C.

#### 3.3.1. Thrombin Inhibitory Assay

Thrombin inhibitory assays were performed following the procedure of Anas *et al.* [22,40,41] using 1 mg/mL and 100 µg/mL concentrations with H_2_O, 50% EtOH or 100% EtOH as solvents. The final concentration in each assay was 100 µg/mL and 10 µg/mL, respectively. Leupeptin was used as a positive control from Peptide Institute, Osaka, Japan. The *Bz*-Phe-Val-Arg·pNA HCl was purchased from Bachem AG (Bubendorf, Switzerland) and used as a substrate. Solvents H_2_O, 50% EtOH, and 100% EtOH were used as negative controls. Pure compounds were tested at a final concentration of 1 µg/mL unless otherwise indicated.

#### 3.3.2. FVIIa and FVIIa-sTF Assays

##### Preparation of l-α-Cephalin or 3-*sn*-Phosphatidylethanolamine Buffer

The fVIIa and fVIIa-sTF assays used l-α-cephalin buffer solution. The fVIIa-sTF assay was performed following the procedure by Nakagura *et al.* [42] with modification. The l-α-cephalin as buffer solution was prepared as follows: Buffer (A): Five hundred milliliters (500 mL) of water was added to 6.057 g of Tris (hydroxymethyl)aminomethane (Nacalai Tesque, Kyoto, Japan) to make 100 mM Tris-HCl solution; 4.383 g NaCl (Nacalai Tesque) was added to the resulting solution to make 100 mM NaCl, and 500 mg bovine serum albumin (BSA) (Sigma, A7284, St. Louis, MO, USA) was added. The pH was adjusted to 7.40; Buffer (B): A 200 mL of Buffer A was added to 0.3329 g of CaCl_2_ (Nacalai Tesque). The resulting solution (Buffer B) was adjusted to pH 7.48 before it was stored at 4 °C in preparation for the next day experiment. A 30 µg/mL 3-*sn*-phosphatidylethanolamine from the bovine brain (Sigma, USA) or l-α-cephalin was added to Buffer B on the day of the experiment.

##### FVIIa Assay

The 80 µL 3-*sn*-phosphatidylethanolamine buffer, 50 µL of 100 mM fVIIa enzyme in a buffer, and 20 µL of sample solution were dispensed in each well of a 96-well plate (Iwaki: 3881-096, Tokyo, Japan). The 96-well plate with the solution was pre-incubated at 37 °C for 5 min separately together with 1 mM of Chromozyme *t*-PA (*N*-Methylsulfonyl-d-Phe-Gly-Arg-4-nitranilide acetate), from Roche Diagnostics (Mannheim, Germany), dissolved in water as a substrate. The 50 µL of the substrate was added, and the mixture was agitated to start the reaction. The absorbance was noted at 405 nm using Thermo Scientific Multiskan FC microplate photometer until favorable binding was observed.

##### FVIIa-sTF Assay

The same buffer preparation for fVIIa assay was used for the fVIIa-sTF inhibitory assay. The fVIIa: sTF ratio was 0.30 µg/mL: 0.39 µg/mL, and was prepared in Section 3.3.2.

##### Preparation of FVIIa Enzyme

The human factor VIIa (HFVIIa) enzyme, purchased from Enzyme Research Laboratories, South Bend, IN, USA, was added and adjusted with 20 mM Tris-HCl/0.1 M NaCl/pH 7.4. The final enzyme concentration should be 95.06 µg/mL. The 100 µL enzyme solutions were stored in plastic cryogenic vials (Iwaki: 2712-002, Tokyo, Japan) at −80 °C until use. The fVIIa enzyme, 95.06 µg/mL, and 100 µL volume solution was added to 7.822 mL of 3-*sn*-phosphatidylethanolamine buffer on the assay preparation.

##### Preparation of Soluble Tissue Factor (sTF or F3-28H)

The sTF or Recombinant Human Soluble Tissue Factor (F3-28H) or Human F3 was purchased from Creative Biomart, Shirley, NY, USA. The sTF was added with 10 mM PBS, pH 7.4, to make 1 mM (25.624 µg/mL), and transferred in 300 µL volumes in plastic cryogenic vials (Iwaki: 2712-002, Tokyo, Japan), stored at −80 °C until use. The sTF solution (25.624 µg/mL, 300 µL) was added to 4.7 mL of the 3-*sn*-phosphatidylethanolamine buffer in an amber bottle before use.

### 3.4. FVIIa-sTF Assay Procedure

The 30 µL buffer, 100 µL fVIIa-sTF, and 20 µL sample solutions were added to a well in a 96-well plate. The solution was pre-incubated at 37 °C for 5 min, together with 1 mM Chromozym *t-*PA in water as a substrate. A 50-µL substrate was added to start the reaction, agitated, and the absorbance was monitored at 405 nm using Thermo Scientific Multiskan FC microplate photometer. The initial and final readings were noted for 40 min.

### 3.5. LC-MS Preparation of Samples and Determination of fVIIa-sTF Active Compounds 

Acetonitrile (99.8% purity) was purchased from Necalai Tesque, Ultrapure Water (LC/MS grade), and Formic Acid (abt. 99%, LC/MS grade) was purchased from Wako Pure Chemical Industries, Ltd., Osaka, Japan. The reversed-phase C18 (ODS) methanol fractions, which were positive for fVIIa-sTF assays, were subjected to LC-MS and dereplicated to know the active compounds present.

One hundred microliters (100 µL) of 100 µg/mL from an EtOH solution of positive ODS fractions was transferred to a small vial. The EtOH solution was evaporated *in vacuo* at 40 °C before adding 100 µL of 10% MeCN to make up 100 µg*/*mL solution for LC-MS analysis.

The LC-MS analysis was performed using Thermo Finnigan LCQ deca XP Plus LCMS analytical instrument with Agilent 1100 Series capillary liquid chromatography system. The samples were analyzed using a solvent gradient from 10% MeCN with 0.1% HCOOH to 100% MeCN with 0.1% HCOOH over 60 min. The analysis was done using reversed phase super ODS (TSK-gel, TOSOH Bioscience, Tokyo, Japan) 50 × 2 mm column, with flow rate 0.2 mL/min, 30 °C column oven, with 200 °C capillary temperature, and UV detection at 220 nm. Solvent optimization of *M. aeruginosa* NIES-89 40% MeOH fraction used gradient elution from 10% MeCN with 0.1% HCOOH to 15% MeCN with 0.1% HCOOH over 60 min using the aforementioned conditions and parameters. The LC-MS data were processed in Xcalibur Qual Browser *ver.* 1.2–1.3. The total ion chromatogram (TIC) and extracted ion chromatogram (EIC) were treated, and peaks were identified for the probable compounds present.

## 4. Conclusions

This research paves a new avenue for toxic *Microcystis* study on its role in medical research. We deduce the importance of serine protease inhibitory peptides aeruginosins from toxic *Microcystis* strains and relate it to the blood coagulation cascade using the LC-MS technique. Argal-containing aeruginosins are potent fVIIa-sTF inhibitors, which could be found in 40% to 80% MeOH ODS fractions in the study. Aeruginosins are potent fVIIa-sTF inhibitors, and we have detected six aeruginosins by LC-MS. The 40% MeOH fraction of *M. aeruginosa* NIES-89 containing a mixture of aeruginosins 89 A (**26**) and B (**27**) displays an EC_50_ value of 7.123 µg/mL for fVIIa inhibitory assay and a thrombin inhibitory activity of 0.010 µg/mL. The aeruginosin 89 A (**26**)/B (**27**) has a dual inhibitory activity against thrombin and fVIIa with 0.001 thrombin/fVIIa inhibition ratio. We need to develop or increase the thrombin/fVIIa ratio for aeruginosin by subjecting it to a structure–activity relationship (SAR) study in the future. Increasing the thrombin/fVIIa ratio could make aeruginosin more specific to fVIIa, which could be done by peptide modification. Future directions of this research aim to establish the structure-activity relationship (SAR) study of different aeruginosins present in this paper. This research is our preliminary study for aeruginosins as probable fVIIa-sTF inhibitors of the blood coagulation cascade. We aim at establishing the concrete fVIIa-sTF scaffolds, which will result in less bleeding and bleeding complications from cyanobacteria, specifically *Microcystis*, as our future research. We need to develop a new drug that could inhibit fVIIa with less bleeding and bleeding complications in the future.
